# Supplementary figures and images for: Building an Interoperable Rare Disease Multi-omic Resource: The GREGoR Data Model and Dataset
Source: bioRxiv. 2026 May 19:2026.05.15.725546. Preprint. [Version 1] doi: 10.64898/2026.05.15.725546 (PMC13228420; doi:10.64898/2026.05.15.725546)

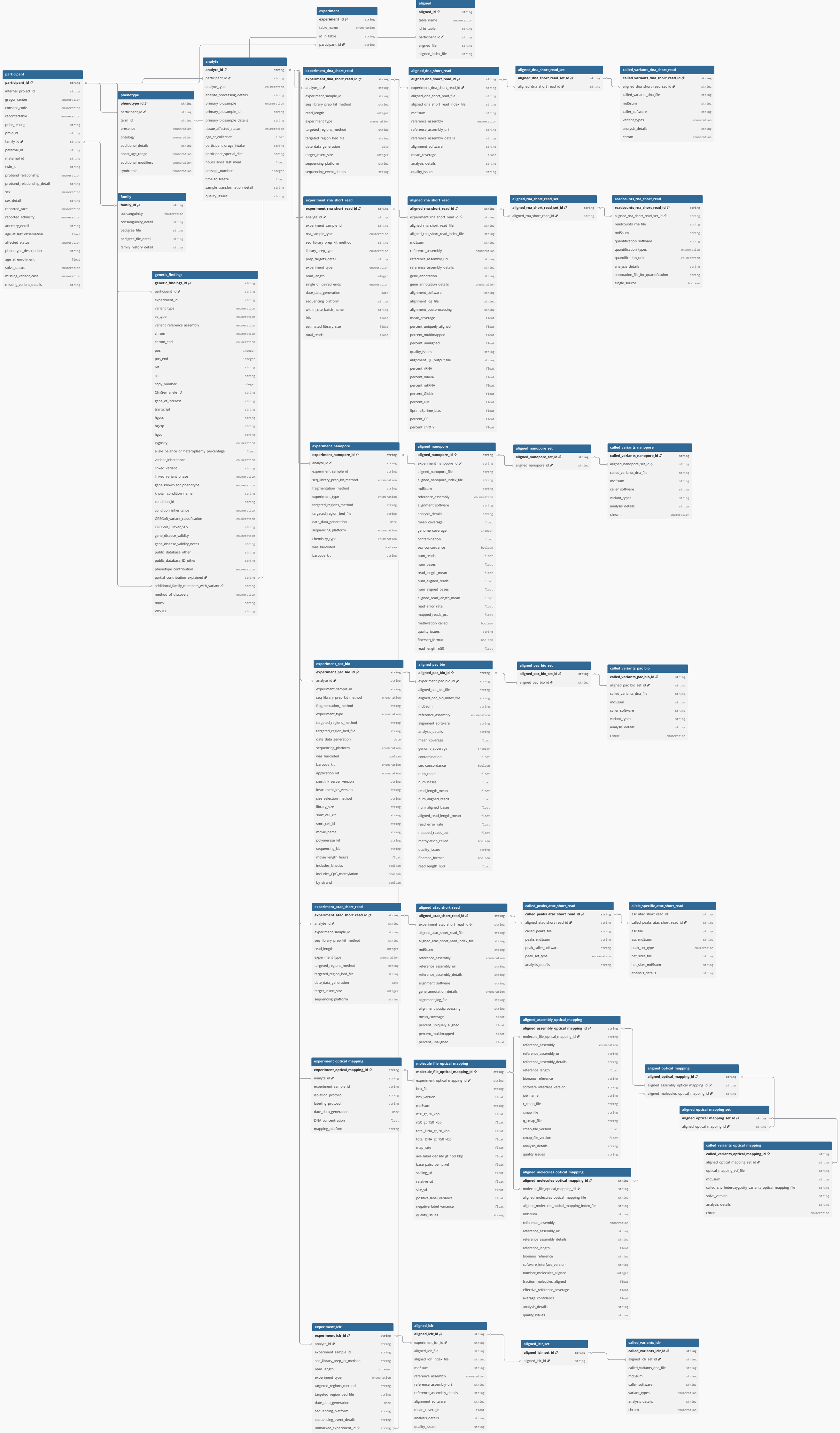

Supplement: Supplement 2 — Document S2: Tables of the GREGoR Data Model .pdf file follows [file media-2.pdf]
