# Supplementary material for: Building an Interoperable Rare Disease Multi-omic Resource: The GREGoR Data Model and Dataset
Source: bioRxiv. 2026 May 19:2026.05.15.725546. Preprint. [Version 1] doi: 10.64898/2026.05.15.725546 (PMC13228420; doi:10.64898/2026.05.15.725546)
Supplement: Supplement 3 — Document S3: Tables S1–S3 (Solve definitions, Variant Interpretation, Completeness of Optional Tables ) .pdf file follows [file media-3.pdf]

Table S1. Solve Status Definitions

Classification	Definition
**Solved**	Pathogenic or likely pathogenic variant(s) with the appropriate inheritance pattern and strong phenotype concordance in a gene with at least moderate evidence of disease association.
**Partially Solved**	Variant(s) explain only a subset of the observed phenotype.
**Probably Solved**	Variant(s) with strong but incomplete evidence, such as a high-level VUS, uncertain phase, or imperfect phenotype match, in genes with at least moderate or near-moderate gene-disease validity.
**Unsolved**	No variant(s) meet criteria for likely causality, including recessive cases with a missing second allele, low-confidence variants, or candidate genes with limited gene-disease evidence.
**Unaffected**	Individuals without the disease phenotype under investigation.

Table S2. Variant Interpretation by Inheritance Model

Dominant Model

Variant Evidence	Phenotype match	Classification
P/LP	Highly specific and consistent	Solved
P/LP	Consistent	Solved
P/LP	Imperfect	Probably Solved
VUS-high	Specific and consistent	Probably Solved
VUS-high	Imperfect	Unsolved
VUS-mid or lower	Any	Unsolved

Recessive Model

Variant Evidence	Phase	Phenotype match	Classification

P/LP + P/LP	Known	Specific and/or consistent	Solved
P/LP + P/LP	Unknown	Specific and/or consistent	Solved (if compelling)
P/LP + VUS-high	Any	Highly specific and/or consistent	Probably Solved
VUS-high + VUS-high	Known	Specific and/or consistent	Probably Solved
Any	Any	Inconsistent	Unsolved
VUS-mid or lower + VUS-mid or lower	Any	Any	Unsolved
Single P/LP (missing allele)	—	Any	Unsolved

Table S3. Completeness of Optional Fields Across Data Tables in the GREGoR Data Model

Table	Mean	Median	STD	Min	Max	n
aligned_atac_short_read	0.67	1.0	0.5	0.0	1.0	9
experiment_dna_short_read	0.64	0.92	0.43	0.0	0.98	8
experiment_rna_short_read	0.55	0.63	0.32	0.0	1.0	9
called_variants_nanopore	0.52	0.47	0.46	0.08	1.0	3
experiment_atac_short_read	0.5	0.5	0.53	0.0	1.0	8
readcounts_rna_short_read	0.5	0.5	0.58	0.0	1.0	4
genetic_findings	0.48	0.55	0.4	0.0	1.0	33
called_variants_pac_bio	0.46	0.38	0.5	0.0	1.0	3
aligned_nanopore	0.46	0.49	0.13	0.0	0.5	14
experiment_nanopore	0.4	0.3	0.43	0.0	0.98	8
called_variants_dna_short_read	0.33	0.0	0.58	0.0	1.0	3
participant	0.32	0.3	0.3	0.0	0.88	14
phenotype	0.25	0.06	0.43	0.0	1.0	5

experiment_pac_bio	0.22	0.0	0.33	0.0	1.0	19
aligned_dna_short_read	0.18	0.06	0.29	0.0	0.69	5
aligned_pac_bio	0.16	0.19	0.08	0.0	0.29	16
aligned_rna_short_read	0.14	0.0	0.18	0.0	0.66	19
analyte	0.05	0.0	0.14	0.0	0.5	12
family	0.03	0.03	0.04	0.0	0.07	4

n denotes the number of optional fields in the corresponding data table.
